# Transcriptome changes and polymyxin resistance of acid-adapted *Escherichia coli* O157:H7 ATCC 43889

**DOI:** 10.1186/s13099-020-00390-5

**Published:** 2020-12-01

**Authors:** Daekeun Hwang, Seung Min Kim, Hyun Jung Kim

**Affiliations:** 1 Research Group of Consumer Safety, Korea Food Research Institute, Wanju, Jeollabuk-do 55365 Republic of Korea; 2 Department of Food Biotechnology, University of Science and Technology, Daejeon, 34113 Republic of Korea; 3 Department of Human Ecology, Korea National Open University, Seoul, 03087 Republic of Korea

**Keywords:** *E. coli* O157:H7, Polymyxin resistance, Acid adaptation, Whole genome sequencing, Transcriptome analysis

## Abstract

**Background:**

Acid treatment is commonly used for controlling or killing pathogenic microorganisms on medical devices and environments; however, inadequate acid treatment may cause acid tolerance response (ATR) and offer cross-protection against environmental stresses, including antimicrobials. This study aimed to characterise an *Escherichia coli* strain that can survive in the acidic gastrointestinal environment.

**Results:**

We developed an acid-tolerant *E. coli* O157:H7 ATCC 43889 (ATCC 43889) strain that can survive at pH 2.75 via cell adaptation in low pH conditions. We also performed RNA sequencing and qRT-PCR to compare differentially expressed transcripts between acid-adapted and non-adapted cells. Genes related to stress resistance, including *kdpA* and *bshA* were upregulated in the acid-adapted ATCC 43889 strain. Furthermore, the polymyxin resistance gene *arnA* was upregulated in the acid-adapted cells, and resistance against polymyxin B and colistin (polymyxin E) was observed. As polymyxins are important antibiotics, effective against multidrug-resistant gram-negative bacterial infections, the emergence of polymyxin resistance in acid-adapted *E. coli* is a serious public health concern.

**Conclusion:**

The transcriptomic and phenotypic changes analysed in this study during the adaptation of *E. coli* to acid environments can provide useful information for developing intervention technologies and mitigating the risk associated with the emergence and spread of antimicrobial resistance.

## Background

Acid treatment is commonly used for the control or elimination of pathogenic microorganisms on the surface of medical devices or in environments, as well as in treatment of wastewater and food, as most microorganisms, including pathogenic bacteria, grow optimally at a pH range of 5–9 [1–3]. For instance, in pharmaceutical and medical environments, hypochlorous acid is used as an antimicrobial agent against a wide range of microorganisms causing wound infections [1]. A nitrous acid as a disinfectant for wastewater for 48 h treatment [4], and a hydrochloric acid (HCl) and organic acids composite was commercially used to spray on meat [5], and a 4 log reduction in *E. coli* O157:H7 and *Listeria monocytogenes* abundance on the lettuce leaf combined with chlorinated water adjusted to pH 2.5 [6] has a synergistic effect.

However, improper or sub-lethal application of acids can induce acid tolerance response (ATR), which contributes to the survival of infectious foodborne pathogens such as enterohaemorrhagic *Escherichia coli* (EHEC), *Salmonella, Shigella* spp., and *Listeria monocytogenes* in acidic environments, including the human gastrointestinal [2]. The risk of infection, including foodborne illness, may increase if the pathogen survives under extreme acid stress (pH 2.0–3.0), as is found in the gastric fluid or following acid treatment of medical devices [7]. In our previous study, 22–33% of commensal *E. coli* food isolates survived in gastric pH conditions of the Korean population, and thus the antimicrobial resistance gene can be transferred from the surviving cells to resident microbiota in the human gut [8]. However, ATR can cause many side-effects other than pathogen survival in acidic environments. For instance, that cross-protective properties can develop in acid-resistant cells is a typical example: ATR cells were reported to have increased resistance to several stresses including heat, salt, crystal violet, and antimicrobials [9]. ATR can also affect the ability of pathogens to bind to surfaces and form biofilms, by increasing cell-surface hydrophobicity, which correlates with pathogenic adhesion to various surfaces in medical and food environments [10, 11].

These biological variabilities provide a mechanism for foodborne pathogens to survive in changing environmental conditions, and thus, are critical targets for mitigating the risk of infectious disease [12]. The relationship between differential gene expression and phenotypic variability, which is often determined by various omics technology, including genome-wide sequencing and transcript analysis, provides increasingly detailed insights into cellular responses to changing environments [13]. Moreover, analysis of transcriptome changes during the adaptation of foodborne pathogens following exposure to acidic environments can provide useful information for developing management strategies to reduce the risk of infectious diseases. Although ATR of foodborne pathogens is an important issue for public health, studies on gene expression profiles and cross-protection from antimicrobials in the context of ATR of foodborne pathogens are limited.

EHEC strains can cause a spectrum of human diseases, ranging from watery diarrhoea and bloody stool to serious extraintestinal complications such as haemolytic uremic syndrome. Among the two identified strains harbouring the Shiga toxin-producing gene, a clinical isolate of ATCC 43889 was selected herein, for analysing the transcriptome changes during ATR, as this isolate was originally acid-sensitive, however, was reported to adapt to acidic conditions [14–16]. In contrast, the well-known EDL933 (ATCC 43895) strain is already acid-resistant [2, 14, 15]. Considering the importance of ATR in foodborne pathogens for public health, analysis of the transcriptome and phenotypic changes during the adaptation of such pathogens to acidic environments can provide information that can be useful for developing intervention technologies and mitigating the risk owing to ATR pathogens. Therefore, in the current study, we aimed to develop an acid-resistant ATCC 43889 strain via cell adaptation in a sub-lethal acidic environment for 100 h. We conducted RNA sequencing (RNA-seq) and quantitative reverse transcription-polymerase chain reaction (qRT-PCR) to compare differentially expressed transcripts between the acid-adapted and non-adapted bacteria. We also performed de novo whole genome sequencing (WGS) of ATCC 43889, which has not been reported previously. Finally, antimicrobial resistance and biofilm formation of the ATR strains were investigated as changes in phenotypic characteristics related to cross-protection from antimicrobials of the ATR strain.

## Results

### Acid adaptation of *E. coli*

**Fig. 1:**
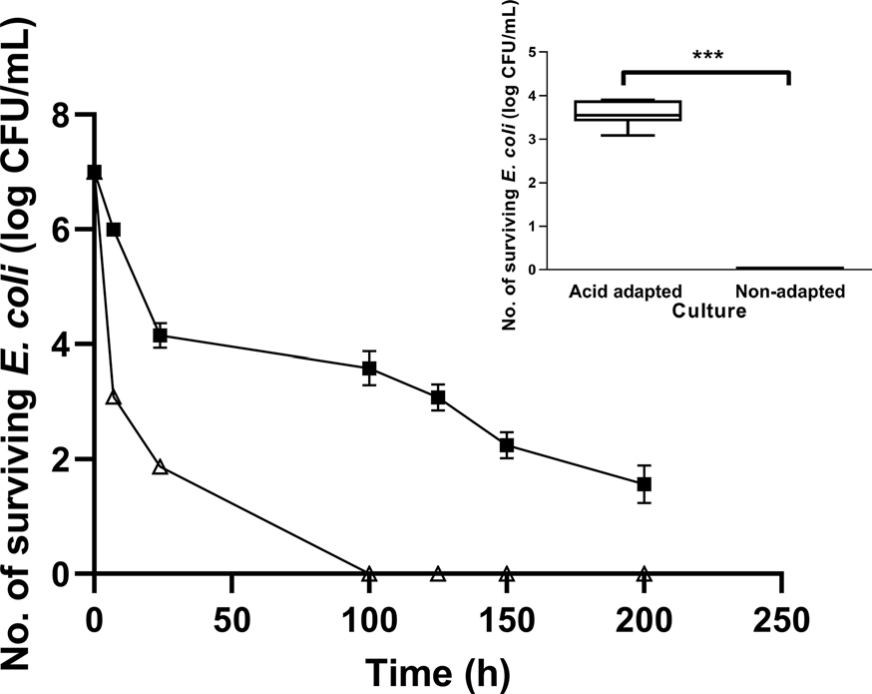
Survival of acid-adapted and non-adapted *Escherichia coli* in pH 2.75 of M9 media. ■, acid-adapted cells; △, non-adapted cells. The acid-adapted and non-adapted *E. coli* were grown in M9 media, pH 2.75, for 200 h. When the pH was 2.75, the acid-adapted cells survived for > 100 h of incubation. The inset figure shows the number of surviving acid-adapted *E. coli* after 100 h incubation with statistical significance (*P* < 0.001)

The pH change in M9 after *E. coli* inoculation was less than 0.02 from the original pH adjusted with 5 N HCl. During the 100 h incubation, no pH changes were observed in two independent experiments. Upon serially decreasing the pH from 4, the lowest pH at which the acid-adapted ATCC 43889 was able to survive for 100 h was 2.75. The cells surviving at pH 2.75 for 100 h were termed acid-adapted ATCC 43889. Figure 1 shows the fates of acid-adapted and non-adapted ATCC 43889 in M9 media of pH 2.75 for 200 h. The number of surviving acid-adapted cells was 3.58 ± 0.30 log CFU/mL at pH 2.75 for 100 h. The acid-adapted cells survived even after 200 h incubation at pH 2.75. However, the non-adapted cells did not survive for 100 h under the same pH conditions. The number of cells surviving after 100 h of incubation at pH 2.75 (p < 0.001) differed significantly for acid-adapted and non-adapted cells, which is shown in the inset of Fig. 1.

### General features of WGS

**Fig. 2:**
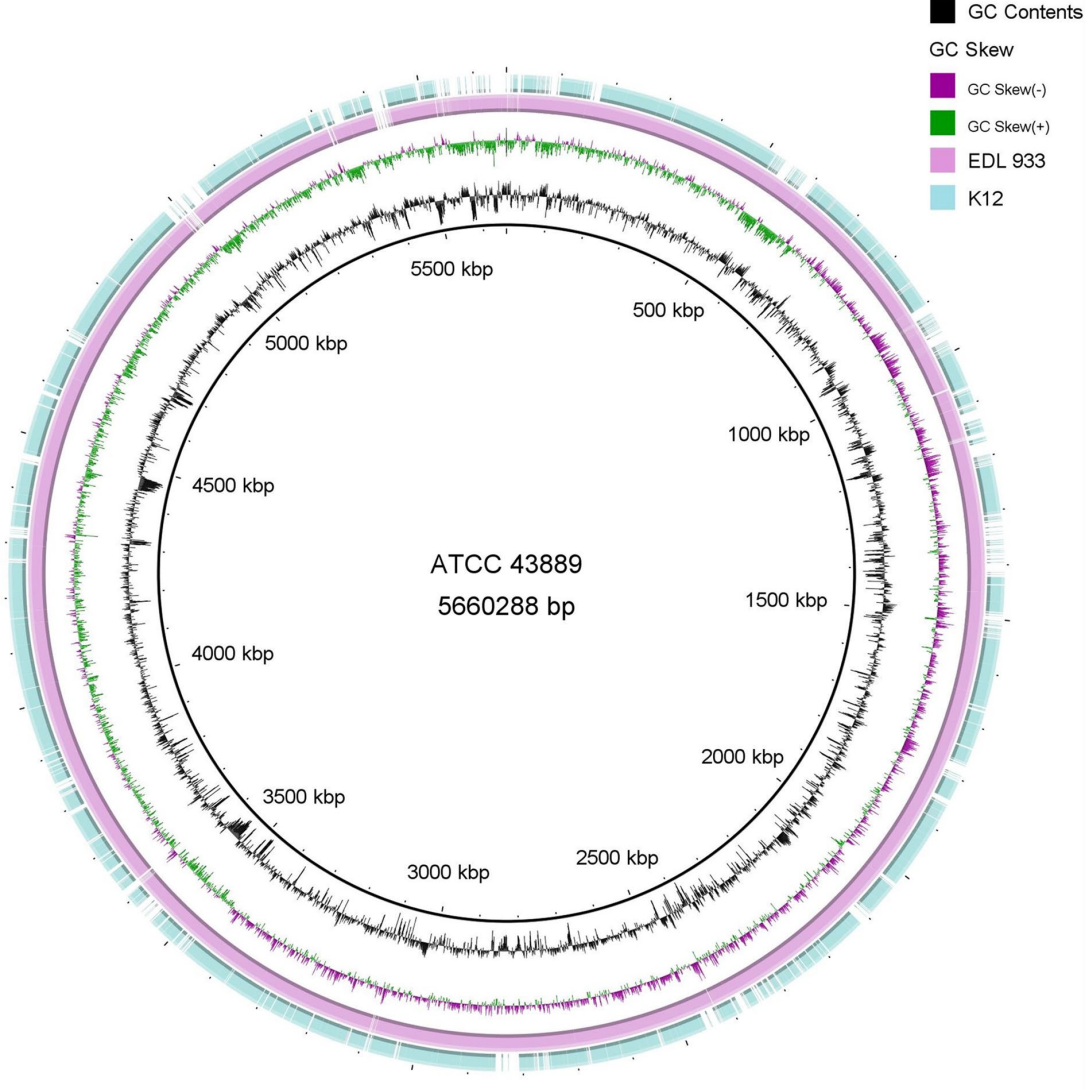
Comparative genomics of *Escherichia coli* genomes. Comparison of the genomes of *E. coli* strains using the ATCC 43889 strain as the reference. Inside out, the first ring represents the ATCC 43889 DNA strand. The second ring represents the ATCC 43889 GC content. The third ring shows the ATCC 43889 GC skew. The outer rings represent the nucleotide sequence of the corresponding DNA regions of the following *E. coli* genomes in different colours against the reference genome (ATCC 43889): EDL933 (pink) and K-12 MG1655 (light blue). The white gaps and low similarity areas in intraspecies genome comparisons show < 50% identity with the BLASTn matches. The genome map was created using BRIG (Blast Ring Image Generator)

Owing to a lack of available published WGS data for ATCC 43889, to map the RNA-seq data, we analysed the WGS of ATCC 43889. The detailed genomic sequences for ATCC 43889 are provided in Additional file 1: Tables S1, S2, Figures S1 and S2. The analysed genome sequence of ATCC 43889 was determined to be 98 and 76% similar to that of EDL933 (acid-resistant pathogenic *E. coli*) and K-12 (commensal *E. coli*), respectively. The white gaps and area of low homology identified in intraspecies genome comparisons indicate < 50% identity with BLASTn matches. Moreover, ATCC 43889 contained unique regions at 5,400 kb, with no matching genes reported for EDL933 or K-12 strains (Fig. 2). Within this region were 20 prophage-related unnamed genes, and 16 genes that encode hypothetical proteins, *yegS,* and *hin*.

### RNA-seq and differential expression analysis

Total RNA was purified from the acid-adapted and non-adapted cells to identify the differentially expressed genes (DEGs). The RNA-seq results were mapped to those of WGS with ATCC 43889 as a reference strain (Additional file 1: Table S3). In total, 59 genes were differentially expressed between the acid-adapted and non-adapted strains. Twenty genes were upregulated and 49 were downregulated in the acid-adapted strain; the most differentially expressed genes are shown in Table 1, whereas the remainders are shown in Additional file 1: Table S4. The most highly upregulated named gene was *kdpA,* with a log_2_ 3.23-fold increase. The upregulated genes were associated with stress resistance and their expression levels were stress-induced; the *bhsA* genes encode multiple stress resistance proteins, and two unnamed genes (*RS06135* and *RS08170*) encode stress-induced bacterial acidophilic repeat motif protein. However, although the p-value was > 0.05, the polymyxin resistance gene *arnA* was also upregulated, suggesting that acid-adapted *E. coli* was cross-protected from the antimicrobial. The genes downregulated in this study mainly encoded a group of proteins associated with maltose metabolism such as *lamB*, *malK*, and *malE.*

**Table 1:** Differentially expressed genes in acid-adapted *E. coli* O157:H7 ATCC 43889 compared to corresponding non-adapted strains

Regulation	Rank	length (bp)	Fold change log_2_ ratio	p-value	Gene symbol	Product	Functional category^a^
Up	1	189	4.95	0.02	*RS05560*	Cell division inhibition protein DicB	Biological process: single-organism process, cellular process, biological regulation
	2	564	4.95	0.02	*RS06705*	Terminase small subunit	Biological process: cellular process
	3	207	3.72	0.08	*RS05320*	Phage tail protein	Cellular component: membrane,
	4	2124	3.72	0.08	*RS27375*	Terminase	Molecular function: binding
	5	366	3.61	0.02	*RS28370*	HNH endonuclease	Biological process: metabolic process
							Molecular function: catalytic activity
	6	1674	3.23	0.01	*kdpA*	Potassium-transporting ATPase A chain	Biological process: localization, organism process
							Cellular component: membrane part, membrane, cell part
							Molecular function: transporter activity, binding, catalytic activity
	7	147	3.23	0.02	*RS06750*	Hypothetical protein	No hit
	8	261	2.63	0.03	*bhsA*	Multiple stress resistance protein BhsA precursor	Biological process: metabolic process, single-organism process, cellular process, response to stimulus
							Cellular component: membrane part, cell part
	9	108	2.61	0.03	*RS08170*	Stress-induced bacterial acidophilic repeat motif protein	Biological process: unclassified
							Cellular component: unclassified
							Molecular function: unclassified
	10	1491	2.54	0.03	*patA*	Putrescine aminotransferase	Biological process: metabolic process, cellular process
							Cellular component: cell part
							Molecular function: binding, catalytic activity
	11	243	2.39	0.05	*RS16110*	Polysaccharide production threonine-rich protein	No hit
	12	468	2.27	0.08	*RS27360*	Endopeptidase	Biological process: cellular process
	13	183	2.20	0.07	*RS06135*	Stress-induced bacterial acidophilic repeat motif protein	Biological process: unclassified
							Cellular component: unclassified
							Molecular function: unclassified
	14	309	2.16	0.09	*RS04915*	Phage tail assembly protein T	No hit
	15	141	2.15	0.08	*RS25935*	Hok/gef family protein	Cellular component: membrane
	16	430	2.15	0.08	*ibpB*	Small heat shock protein IbpB	Biological process: response to stimulus
							Cellular component: cell part
							Molecular function: binding
	17	96	2.09	0.09	*RS03750*	Hypothetical protein	No hit
	18	132	2.07	0.11	*RS26305*	Hypothetical protein	No hit
	19	1983	2.05	0.10	*arnA*	Bifunctional polymyxin resistance protein ArnA	Biological process: metabolic process, response to stimulus, single-organism process, cellular process
							Molecular function: binding, catalytic activity
	20	213	2.03	0.10	*RS00040*	Hypothetical protein	No hit
Down	1	222	−6.75	0.01	*RS26915*	TraR/DksA family transcriptional regulator	Biological process: biological regulation
	2	246	−4.52	0.07	*RS27335*	Hypothetical protein	No hit
	3	366	−4.21	0.01	*RS03415*	HNH endonuclease	Biological process: metabolic process
							Molecular function: catalytic activity
	4	1341	−4.20	0.00	*lamB*	Maltoporin precursor	Biological process: localization, response to stimulus, multi-organism process, single-organism process, cellular process
							Cellular component: membrane part, membrane, macromolecular complex, cell part
							Molecular function: molecular transducer activity, transporter activity, binding
	5	921	−3.17	0.01	*malM*	Maltose operon protein MalM	Biological process: localization, single-organism process
							Cellular component: cell part
	6	750	-3.03	0.01	*invF*	Invasion protein InvF	Biological process: biological regulation
							Molecular function: nucleic acid binding transcription factor activity
	7	351	−3.03	0.01	*RS15960*	Hypothetical protein	Biological process: response to stimulus
	8	228	−2.92	0.07	*RS03410*	DUF3950 domain-containing protein	No hit
	9	285	−2.92	0.02	*RS15965*	Hypothetical protein	No hit
	10	1116	−2.81	0.02	*malK*	Maltose/maltodextrin import ATP-binding protein MalK	Biological process: localization, single-organism process
							Cellular component: membrane part, membrane, macromolecular complex, cell part
							Molecular function: transporter activity,
							binding, catalytic activity
	11	333	−2.79	0.02	*RS14560*	DUF2686 family protein	No hit
	12	360	−2.78	0.02	*RS15860*	Hypothetical protein	No hit
	13	1191	−2.72	0.02	*malE*	Maltose-binding periplasmic protein precursor	Biological Process: localization, response to stimulus, single-organism process, - locomotion, cellular process
							Cellular component: membrane part, macromolecular complex, cell part
							Molecular function: transporter activity, binding
	14	1416	−2.71	0.02	*tnaA*	Tryptophanase	Biological process: metabolic process, single-organism process, cellular process
							Cellular component: membrane, cell part
							Molecular function: binding, catalytic activity
	15	927	−2.63	0.03	*coaA_1*	Pantothenate kinase	Biological process: metabolic process, single-organism process, cellular process
							Cellular component: cell part
							Molecular function: binding, catalytic activity

^a^Functional categories were based on GO analysis classifications were based on annotation from the *E. coli* best-hit gene models (E value ≤ 10^–5^) and GO analysis search using the BLAST2GO program

### Validation of gene expression

**Fig. 3:**
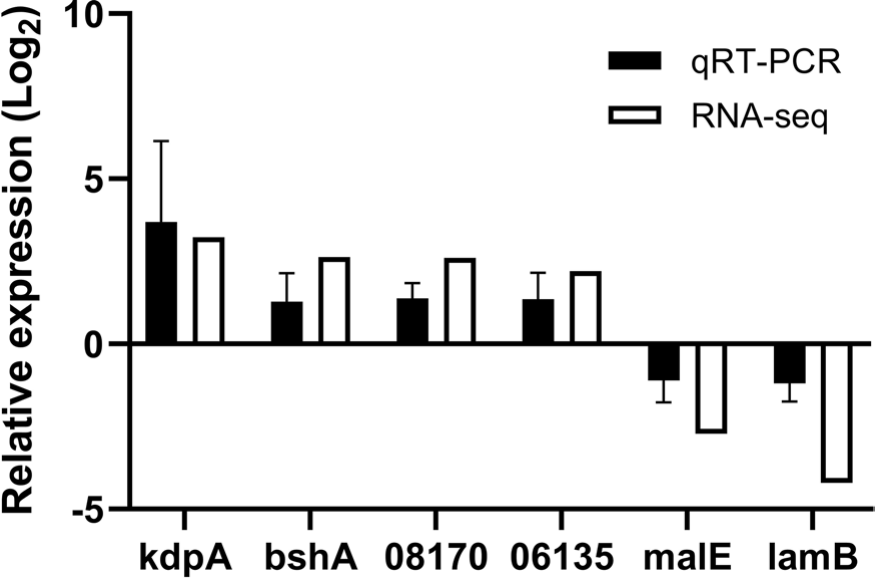
Validation of RNA-seq data of selected genes using qRT-PCR. The relative expression levels of selected genes were determined using qRT-PCR. Each column represents the mRNA expression level in acid-adapted *Escherichia coli* relative to that in non-adapted *E. coli*. The expression levels are the mean ± SEM of the results from at least three independent experiments

To validate the RNA-seq results, we performed qRT-PCR for six selected genes, *kdpA*, *bshA*, *6135*, *8170*, *lamB*, and *malE*, which showed significantly altered expression in RNA-seq (Table 1). The 16S rRNA housekeeping gene expression level did not differ for both acid adapted and non-adapted cells (Ct value for acid-adapted cells were 5.35 ± 0.73 and non-adapted cells were 5.22 ± 0.69). The results of qRT-PCR are presented as means with standard deviations. A similar pattern of upregulation and downregulation was observed in both RNA-seq and qRT-PCR analyses (Fig. 3). However, the log_2_ fold-changes observed using qRT-PCR were slightly lower than those from RNA-seq. For example, the results of RNA-seq showed that *lamB* expression in acid-adapted cells was log_2_ 4.20-fold lower than that in non-adapted cells, whereas this difference was only log_2_ −1.19 ± 0.55-fold in the qRT-PCR results. Alternatively, for *kdpA*, qRT-PCR showed a higher fold-change in expression than did RNA-seq with the latter showing a log_2_ 3.10-fold change, and qRT-PCR showed a log_2_ 3.69 ± 2.44-fold change. However, similar expression levels were observed for *bshA_3* by RNA-seq (log_2_ 2.05-fold change) and qRT-PCR (log_2_ 1.64 ± 0.48-fold change) analysis.

### Phenotype variation

**Fig. 4:**
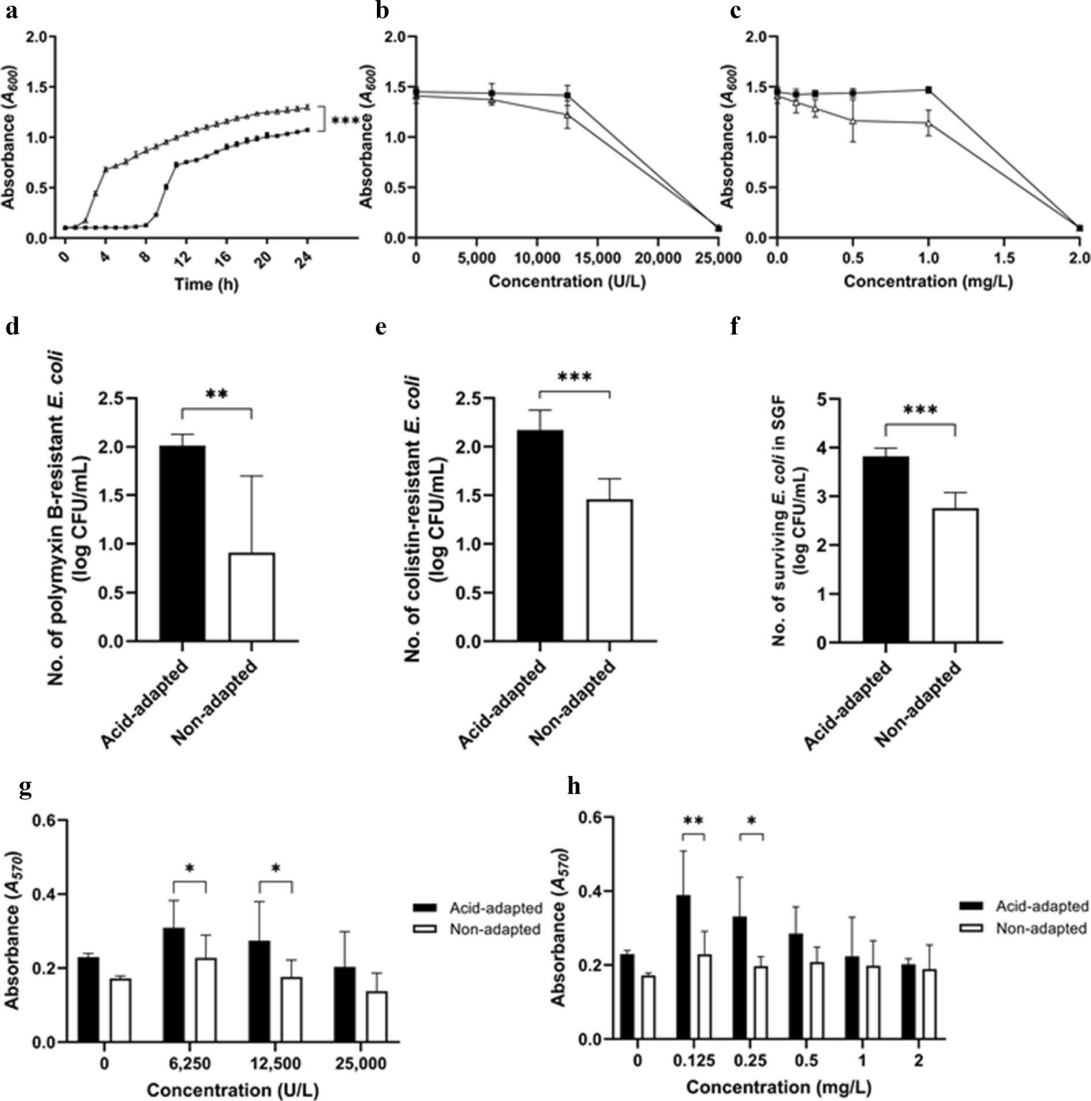
Growth rate, antimicrobial resistance, and biofilm formation of acid-adapted and non-adapted cells. p < 0.05 (*), 0.01 (**), and 0.001 (***). **a** The absorbance of acid-adapted cells was fourfold higher at the lag phase than that of the non-adapted cells in tryptic soy broth. The maximum population density of acid-adapted cells was lower than that of non-adapted cells (p < 0.001) ■, acid-adapted cells; △, non-adapted cells. **b** MIC of polymyxin B for ATCC 43889. ■, acid-adapted cells; △, non-adapted cells. **c** MIC of colistin for ATCC 43889. ■, acid-adapted cells; △, non-adapted cells. **d** Survival of *Escherichia coli* on MHA with polymyxin B (12,500 U/L). The number of polymyxin B-resistant acid-adapted cells was higher than that of non-adapted cells (p < 0.01). **e** Survival of *E. coli* on MHA with colistin (1 mg/L). The number of colistin-resistant acid-adapted cells was higher than that of non-adapted cells (p < 0.001). **f** Survival of *E. coli* in simulated gastric fluid for 3 h. The number of surviving acid-adapted cells were 1 log higher than that of non-adapted cells (p < 0.001). **g** Biofilm formation of cells incubated with polymyxin B. **h** Biofilm formation of cells incubated with colistin

The lag phase duration of acid-adapted ATCC 43889 8 h was (fourfold) longer than that of non-adapted cells 2 h with significance. The maximum population density of acid-adapted cells (OD 1.07) was lower than that of non-adapted cells (OD 1.31) (p < 0.01, Fig. 4a), indicating the fitness cost of acid-adapted cells under normal conditions. Moreover, according to the results of the broth microdilution test, the minimal inhibitory concentration (MIC) of ATCC 43889 was 25,000 U/L for polymyxin B and 2 mg/L for colistin, suggesting that this strain has intermediate susceptibility [17]. However, no differences were observed between the MICs of acid-adapted and non-adapted cells (Fig. 4b, c). Further, no colonies were also observed at MIC according to the agar diffusion test of polymyxins. However, at sub-MIC, the acid-adapted *E. coli* showed higher resistance to polymyxin B and colistin than the non-adapted cells (p < 0.01, Fig. 4d, e), as indicated by upregulation of the polymyxin resistance gene. The induced resistance of acid-adapted cells to polymyxins suggests that acid adaptation provides cross-protection from other forms of environmental stress.

The initial number of inoculated bacterial cells in simulated gastric fluid (SGF) was approximately 5 log CFU/mL. After the acid-adapted and non-adapted cells were exposed to the SGF for 3 h, the number of acid-adapted cells (3.82 log CFU/mL) was 1 log higher than that of non-adapted cells (2.75 log CFU/mL) in the SGF (p < 0.001) (Fig. 4f).

Lastly, no significant changes were observed in biofilm formation during the first two days of incubation regardless of the presence of polymyxins. Furthermore, although the expression of *bhsA,* a gene related to biofilm formation, was upregulated in acid-adapted *E. coli,* during the seven days incubation without polymyxins, no significant changes were observed in biofilm formation. Alternatively, at sub-MIC concentrations, significantly higher biofilm formation were observed in acid-adapted cells following seven days of incubation, i.e., when cells were incubated at 6,250 and 12,500 U/L of polymyxin B and 0.125 and 0.25 mg/L of colistin for seven days (Fig. 4g, h).

## Discussion

Exposure of ATCC 43889 cells to acidic environments induces an acid adaptation response. A previous study showed that acid adaptation at approximately pH 5 was required to obtain increased resistance to a sub-lethal pH (pH 3.0) [14, 18]. Furthermore, acid adaptation may result in enhanced protection against lethal heat, a process often referred to as cross-protection owing to exposure of bacteria to acidic conditions. However, information regarding transcriptomic changes in ATCC 43889 adapted under sub-lethal pH conditions is limited. In this study, transcriptomic changes were observed in *E. coli* adapted at the extremely acidic pH of 2.75, which is found in some food items such as vinegar [19]. As the gastric pH in humans ranges from 2.0 to 4.8 depending on the food buffering capacity [20], *E. coli* adapted to pH 2.75 can survive in the human stomach, the first barrier against pathogens that cause foodborne illness, or transfer virulence genes, as well as antimicrobial resistance genes, to gut microbiota or to bacteria in the external environment such as food and/or biofilms [8, 21, 22].

As predicted, the RNA-seq results showed that genes associated with stress regulation were upregulated in acid-adapted cells. In our study, *kdpA* was highly upregulated (log_2_ 3.23-fold) in the acid-adapted cells, compared to that in the non-adapted cells, thus supporting the results of a previous study [23]. *kdpA* encodes a potassium-binding subunit of a potassium-transporting ATPase, which functions to bind and transport potassium ions across the cytoplasmic membrane [24]. Responses to acidic stress involve different cellular mechanisms, such as proton pumps [25]. Martirosov et al. described the H^+^-K^+^ exchanging system, which involves a dicyclohexylcarbodiimide-sensitive exchange of 2 H^+^ from cells for 1 K^+^, and it is carried out through a proton pump [26]. Furthermore, the KdpFABC complex comprises an ion channel, an ion pump, and an ABC transporter. The KdpA protein corresponds structurally to an ion channel [27]. This is essential for the survival of *E. coli* as it blocks H^+^ ions from crossing the cell membrane. Moreover, upregulated *kdpA* was detected in acid-adapted *Salmonella* Typhimurium [28]. In this study, *kdpA* was upregulated in *E. coli* that survived in acidic environments for more than 100 h. Additionally, upregulation of *bhsA* (previously *ycfR*)*,* which encodes a putative outer membrane protein, was also observed. During chlorine treatment, *bhsA* was the most significantly upregulated gene, which encodes a protein directly involved in the cellular transport of metabolites [29, 30]. *bhsA* is also known to significantly induce biofilm formation in *E. coli*, although no significant increase in biofilm formation was observed in the acid-adapted cells in this study.

The growth rate of the acid-adapted cells was lower than that of non-adapted cells under the same conditions, which may result from fitness cost as resistance to environmental changes is often related to reduced bacterial fitness (the ability to survive and reproduce) [31]. Alternatively, reduced growth rate of the acid-adapted cells may be related to downregulation of *lamB*, *malK*, and *malE*, which are associated with maltose metabolism. According to Nuoffer et al., *E. coli* produces glucose intracellularly via phosphorylation of maltose [32]. A previous report showed that the genes regulating maltose metabolism (*malE, lamB)* were highly upregulated when glucose was limited as a nutrient [33]. We observed a decrease in the expression of maltose genes in acid-adapted cells, which is supported by the results of a previous study which reported that expression of *lamb,* encoding the maltoporin precursor, was strongly reduced in various K-12 strains, as observed using SDS-PAGE, when the pH of the growth medium was decreased [34]. Moreover, the expression of *malE*, a gene associated with maltose-binding periplasmic protein maltose receptor, decreased during recovery from acid stress [35]. *malE* is known to be related to alkaline induction [36]. Therefore, downregulation of *lamB* and *malE* may be a hallmark of acid adaptation.

Interestingly, *gadA/B* was not identified in our differential gene expression analysis, which may have been caused by two factors. First, M9 media does not contain glutamate. Previous studies have shown that the glutamate-dependent AR pathway consists of the glutamate decarboxylases *gadA/B* and the glutamate/γ-aminobutyric acid antiporter *gadC*, which showed low activity in the absence of glutamate [37]. Second, *gadA/B* undergoes a stepwise conformational change to its inactive form when the pH increases back to neutral, and the optimal pH for *gadABCEWX* and *ybaS* gene expression is pH 5.5 [38–41]. Gene expression patterns differ following long- and short-term acid adaptation; for example, gene expression of *E. coli* K-12 in glucose-limited media after short-term adaptation was 12.6-fold higher than that after long-term adaptation [42]. These results suggest that gene expression is reduced after long-term adaptation.

Interestingly, in this study, the polymyxin resistance gene *arnA* was upregulated in acid-adapted *E. coli* O157:H7, and the antimicrobial resistance against polymyxin B and colistin confirmed the phenotypic changes in these cells. There is similar report on the development of antimicrobial resistance in the foodborne pathogens (*E. coli*, *S.* Typhimurium, and *S. aureus*) exposed to acidic conditions, i.e., resistance against amikacin, ceftriaxone and trimethoprim for gram negative strains and gentamicin and erythromycin for gram positive strains were developed when the cells are contact with sublethal pH conditions for 24 h [43]. However, the upregulated *arnA* did not affect the MIC of polymyxins in acid-adapted cells. According to Sinel et al., a 14.3-fold upregulation of the gene encoding the quinolone resistant protein resulted in a 2- to 6-fold increase in MIC for *Enterococcus faecium* [44]. Owing to the prevalence of antibiotic resistance and lack of novel classes of antibiotics in the development pipeline, clinical use of polymyxins has significantly increased over the past decades [45]. The most common mechanism of acquired resistance to polymyxins is modification of the bacterial outer membrane lipopolysaccharide. Global epidemiological surveillance studies have reported the occurrence of polymyxin resistance to be common in Enterobacteriaceae, specifically in *Enterobacter* species and in *Acinetobacter baumannii* [46]. Considering the limited number of agents available for treating infections caused by multidrug-resistant gram-negative organisms [46], the emergence of polymyxin resistance, particularly from the cross-protection of acid-adapted EHEC, is of considerable concern. In this study, increased biofilm formation was observed at sub-MIC concentrations of polymyxins, which provides additional protection against environmental stresses including antimicrobials. Similar results on increased biofilm formation in *E. coli* were observed under sub-MIC concentrations of glycopeptide, cyclic peptide, fluoroquinolone, and β-lactam [47].

This study has some limitations; acid adaptation procedure was not a typical, but considering practices in the food industries or medical environments. It is more realistic to alternate exposure to acidic and neutral environments. We only observed phenotypic changes and DEG of acid-adapted *E. coli* O157:H7 but molecular basis was not explored. Thus, studies on the phenotypic changes and DEGs of other types of pathogenic *E. coli* and molecular basis (e.g. gene deletion and/or protein study) of the acid-adapted cells are needed in the future work. However, phenotypic changes in ATR of pathogenic bacteria with relation to gene expression can be incorporated in safety management of infectious pathogens to ensure public health.

## Conclusion

In this study, the ATCC 43889 strain, which was originally acid-sensitive, was adapted under sub-lethal acidic conditions. WGS showed that the genetic sequence was 98% homologous to that of the EDL933 strain, which is an acid-resistant strain. The main findings of the study are as follows; (i) upregulation of the antimicrobial resistance gene *arnA* in the acid-adapted ATCC 43889 strain, which was validated, induced antimicrobial resistance against polymyxin B and colistin; (ii) upregulation of stress-related genes, including *kdp*A and *bsh*A; and (iii) survival of acid-adapted cells in SGF was higher than that of non-adapted cells. The results of genomic, transcriptomic, and phenotypic analyses of the acid-adapted cells have provided information that will be useful for developing safety management strategies to reduce the risk of infectious diseases and for further studies on the antibiotic resistance of acid-adapted pathogens.

## Materials and methods

### Preparation of bacterial strain and acid adaptation

The *E. coli* O157:H7 (Migula) Castellani and Chalmers ATCC® 43889™ strain was obtained from the National Culture Collection for Pathogens (Osong, Korea). The commensal *E. coli* ATCC® 10536™, obtained from the Korean Type Culture Collection (Daejeon, Korea), was used as negative control. Bacteria were inoculated into 5 mL of tryptic soy broth (TSB) (Merck, Darmstadt, Germany) in 17 × 100-mm glass culture tubes at 37 °C for 24 h in a shaking incubator at 140 rpm (SI-600R, Jeio Tech, Korea). A volume of 50 μL of the broth cultures of a stationary phase was subsequently transferred to 5 mL of M9 minimal media containing 0.4% glucose supplemented (MB Cell, Los Angeles, CA, USA) at 37 °C for 24 h with agitation. The cells were harvested, washed, and suspended in phosphate buffered saline (PBS) (Gibco, Rockville, MD) to obtain a stationary phase-adjusted concentration of approximately 10^7^ CFU/mL. For the acid-adapted ATCC 43889 strain, a 200-μL aliquot of the cultures was transferred to 10 mL M9 media without supplement at pH 4. The pH of M9 (control, pH 7.4) was adjusted by adding 5 N hydrochloric acid (Kanto Chemical, Tokyo, Japan; PubChem CID: 313) before inoculation. Next, the inoculated acidic M9 was incubated for 24, 50, 75, and 100 h with agitation without medium change. After incubation, the cultures were serially diluted and the adapted cells were recovered in triplicate on Luria Bertani agar (LB) (Merck) and sorbitol-MacConkey agar (SMAC) (Merck) at 37 °C for 24 h. Following 100 h of incubation in acidic growth media, the surviving bacteria were recovered after incubation on agar plates for 24 h and the colonies were counted. If colonies were recovered on both agar plates, a single colony was picked from SMAC agar and the process was repeated in pH reduced by 0.25 until no colonies were recovered. The pH of M9 media immediately preceding the pH at which no colonies were obtained was considered the final acid-adapted pH. At the final pH, a 200-μL aliquot of acid-adapted and non-adapted cells were transferred to 10 mL of acidic M9 media without supplement and incubated for 200 h with agitation without medium change to analyse survival as presented in Fig. 1. Survival of *E. coli* in acidic M9 media were performed in duplicate and the data presented were obtained from at least three independent experiments.

### DNA isolation, WGS, assembly, and functional annotation

The experimental procedures are described in detail in the Additional file 1.

### Total RNA isolation and RNA-seq

RNA-seq analysis was used to compare gene expression between acid-adapted and non-adapted cells. Total RNAs were isolated from acid-adapted cells that survived at pH 2.75 of M9 medium for 150 h and the corresponding non-adapted cells using a Qiazol lysis reagent (Qiagen GmbH, Hilden, Germany) according to the manufacturer’s instructions. The DNA in each sample was removed using an on-column digestion using RNase-free DNase I (Qiagen).

Following RNA extraction and DNase I treatment, ribosomal RNA (rRNA) was removed using the Ribo-Zero rRNA removal kit (Bacteria) (Illumina, San Diego, CA, USA) and cDNA was synthesised using DNA polymerase I and RNase H (Illumina) according to the manufacturer’s instructions. The quantity and quality of the total RNA was analysed using NanoVue Plus (GE Healthcare, Buckinghamshire, UK) and a 2100 Bioanalyzer (Agilent Technologies, Santa Clara, CA, USA) with an RNA integrity number (RIN) ≥ 8. The purified expression libraries were sequenced (100 base pair (bp) × 2) using a HiSeq 2000 (Illumina) platform. The presented RNA-sequencing data were obtained from an independent experiment and analysed in duplicate.

### Quality control and data analysis of RNA-seq

Sequence read data were analysed with FastQC (v0.10), which provides a modular set of analyses and can rapidly reveal sequence quality [48]. In addition, Trimmomatic (v0.32) was used to trim and crop Illumina (FASTQ) data and remove adapters [49], whereas Bowtie was used to align sequences [50]. DEGs were analysed using edgeR and the p-value was obtained from total count normalisation [51].

An in-house script was used to calculate the reads per kilobase per million (RPKM) for individual transcripts [52]. At least one sample with an RPKM value of zero was excluded from the analysis. The quintile method of normalisation was then applied to reduce systematic bias [53]. Genes with differential expression indicated by an absolute log_2_ (fold change) ≥ 2 were selected.

### qRT-PCR validation

qRT-PCR was performed to confirm the DEGs identified using RNA-seq. The primer sequences of candidate genes/transcripts and a housekeeping gene (16S rRNA) were designed using NCBI Primer BLAST (Additional file 1: Table S5). cDNA was synthesised using the Maxima first strand cDNA synthesis kit for qRT-PCR (Thermo Scientific, Waltham, MA, USA) according to the manufacturer’s instructions. SYBR Green (Thermo Scientific) PCR (20 μL) was performed in duplicate for each sample using 250 ng cDNA and 300 nM each of the forward and reverse primers. Forty cycles of amplification and data acquisition were performed on a PikoReal 96 real-time PCR system (Thermo Scientific) (Additional file 1: Table S5). The 2^−ΔΔCt^ method was used to evaluate the expression levels of each target gene compared to that of the 16S rRNA internal control [54]. All experiments were performed in duplicate and the data presented were obtained from at least three independent experiments.

### Phenotyping for growth rate and antimicrobial resistance

Strains that show differential expression of genes under different conditions may show phenotypic variations. To compare growth rate, the acid-adapted and non-adapted cells were incubated in 5 mL TSB at 37 °C for 24 h with agitation, and the absorbance of the broth cultures was evaluated using a BioTek synergy Mx (BioTek Instruments, Winooski, VT, USA) at 600 nm.

To determine the antimicrobial resistance of acid-adapted and non-adapted cells, the MIC of polymyxins was determined by broth microdilution according to a previous study and recommendation of the joint CLSI-EUCAST polymyxin breakpoints, cation-adjusted Mueller–Hinton Broth (Merck) using concentrations of 400,000 U/L polymyxin B sulfate salt (Sigma; PubChem CID: 9833652) and 8 mg/L colistin sulfate salt (Sigma; PubChem CID: 73090) and serially diluted without surfactants (i.e. polysorbate-80) [55, 56]. Polymyxin agar test was also performed [17]. Muller Hinton agar (MHA) plates were prepared with 12,500 U/L polymyxin B sulfate salt and 1 mg/L colistin sulfate salt, which were then inoculated with 100 μL of 0.5 McFarland suspension and incubated for 20 h at 37 °C. Plates with visible growth of microorganisms was read as positive.

Survival of acid-adapted and non-adapted cells in SGF with pH adjusted to 2.75 was determined. A 100 μL bacterial suspension was inoculated into 10 mL of SGF prepared as previously described [57]. The culture was incubated at 37 °C with agitation for 180 min, then 100 μL of the bacterial suspension was plated in triplicate onto SMAC.

Biofilms were formed in the presence of polymyxin B (0, 6250, 12,500, and 25,000 U/L) and colistin (0, 0.125, 0.25, 0.5, 1.0, and 2.0 mg/L) for 1, 2, and 7 days using the procedure developed by Zhu and Mekalanos [58].

All the above experiments were performed in duplicate and the data presented were obtained from at least three independent experiments.

### Statistical analyses of experimental data

The generated data were analysed for statistical significance using the paired two-tailed Student’s *t*-test of GraphPad Prism 8 (GraphPad Software, Inc., San Diego, CA). P values < 0.05 were considered statistically significant.

## Supplementary information

**Additional file 1: Table S1.** Genomic features of ATCC 43889. **Table S2.** Pfam analysis on whole genome annotation. **Table S3.** Statistics of RNA-seq alignment of acid-adapted cells and non-adapted cells when mapped to the reference genome. **Table S4.** Differentially expressed genes down-regulated in acid-adapted *E. coli* O157:H7 ATCC 43889 compared to corresponding non-adapted using RNA sequencing after rank 16. **Table S5.** Primer sequences for qRT-PCR. **Figure S1.** Subsystem distribution of SEED and Kyoto Encyclopaedia of Genes and Genomes (KEGG). (A) Distribution of genes assigned to 600 SEED subsystems (based on the RAST annotation server). (B) In total, 698 genes were assigned to KEGG distribution (based on BLAST KOALA annotation server). **Figure S2.** Gene Ontology (GO) classification of the 5327 annotated proteins. GO analysis classifications were based on annotation from the *E. coli* best-hit gene models (*E* value ≤ 10^–5^) and GO analysis search using the BLAST2GO programme.

## Data Availability

The WGS project of *E. coli* O157:H7 ATCC 43889 has been deposited in GenBank under the accession number CP015853-4.
