# Supplementary material for: Transcriptome changes and polymyxin resistance of acid-adapted Escherichia coli O157:H7 ATCC 43889
Source: Gut Pathog. 2020 Dec 1;12:52. doi: 10.1186/s13099-020-00390-5 (PMC7709258; doi:10.1186/s13099-020-00390-5)
Supplement: Supplementary file 1 — Additional file 1: Table S1. Genomic features of ATCC 43889. Table S2. Pfam analysis on whole genome annotation. Table S3. Statistics of RNA-seq alignment of acid-adapted cells and non-adapted cells when mapped to the reference genome. Table S4. Differentially expressed genes down-regulated in acid-adapted E. coli O157:H7 ATCC 43889 compared to corresponding non-adapted using RNA sequencing after rank 16. Table S5. Primer sequences for qRT-PCR. Figure S1. Subsystem distribution of SEED and Kyoto Encyclopaedia of Genes and Genomes (KEGG). (A) Distribution of genes assigned to 600 SEED subsystems (based on the RAST annotation server). (B) In total, 698 genes were assigned to KEGG distribution (based on BLAST KOALA annotation server). Figure S2. Gene Ontology (GO) classification of the 5327 annotated proteins. GO analysis classifications were based on annotation from the E. coli best-hit gene models (E value ≤ 10–5) and GO analysis search using the BLAST2GO programme. [file 13099_2020_390_MOESM1_ESM.docx]

**Supplementary information**

**Transcriptome changes and polymyxin resistance of acid-adapted *Escherichia coli* O157:H7 ATCC 43889**

Daekeun Hwang, Seung Min Kim, Hyun Jung Kim^*^

Email address: DH: [ant0040@gmail.com](mailto:ant0040@gmail.com), SMK: [kisie@knou.ac.kr](mailto:kisie@knou.ac.kr), HJK: [hjkim@kfri.re.kr](mailto:hjkim@kfri.re.kr)

*Corresponding author Hyun Jung Kim: [hjkim@kfri.re.kr](mailto:hjkim@kfri.re.kr)**Supplementary Materials and Methods**

**DNA isolation and WGS**

DNA of ATCC 43889 was extracted using the MG^TM^ genomic DNA purification kit according to the manufacturer’s guidelines (Macrogen, Seoul, Korea). The DNA content of the samples was analyzed using the PicoGreen dsDNA quantitation kit (Invitrogen, Carlsbad, CA, USA) in a Victor 3 fluorimeter (PerkinElmer, Waltham, MA, USA). DNA that passed the quality check (> 50 ng concentration, 100 μL volume, and 5 μg total amount) was used for library construction. The genome of ATCC 43889 was sequenced on the third-generation sequencing platform PacBio RSII system (Pacific Biosciences, Menlo Park, CA, USA), where a single 10-kb single-molecule real-time-bell sequencing library (Pacific Biosciences) was constructed.

**Assembly and functional annotation**

The draft genome sequence was assembled de novo using HGAP3 (1). The genome was automatically annotated using Prokka v1.11 (rapid Bacterial Genome Annotation) (2). These annotations were confirmed using BLAST analysis. BRIG was used for generating ring images, and comparative genomic analysis (3) was conducted by comparing ATCC 43889 with another pathogenic *E. coli* EDL933 (4) and the commensal *E. coli* K-12 MG1655 (K-12) (5).

Functional domain annotation was performed using the hmmscan from HMMER v3.1b1 (6) suite to search against Pfam database and the SEED Rapid Annotation using Subsystems Technology (RAST) (7). Some metabolic pathways were classified using Kyoto Encyclopedia of Genes and Genome (KEGG) by KOALA (8). Gene Ontology (GO) analysis was performed utilizing the default parameters of the BLAST2GO software (9).

**Supplementary Results**

**Assembly and functional annotation**

The genome of ATCC 43889 was composed of two circular contigs with a combined length of 5,660,288 bp and the GC contents of the main chromosome and plasmid were 50.5% and 47.8%, respectively. Annotation of this assembly using Prokka annotation identified 5,327 coding sequences (CDSs, excluding 339 pseudo CDSs), 107 tRNAs (excluding 0 pseudo tRNAs), three rRNA operons (5S, 16S, 23S) that included 22 rRNA and 8 non-coding RNAs (Table S1); these results were confirmed using the NCBI Prokaryotic Genome Annotation Pipeline (https://www.ncbi.nlm.nih.gov/genome/annotation_prok/). We assigned protein-coding genes to SEED subsystems; the number of CDSs were 5,724, and 600 SEED subsystems were determined. Furthermore, 3,126 CDSs were in subsystems (55%), and 2,598 CDSs were non-hypothetical (Fig. S1A). In KEGG annotation, 698 genes were annotated to the KEGG database (Fig. S1B), and the total categorized gene number was 1,226. The annotated gene numbers and total categorized gene numbers differed, as some genes belonged to multiple categories (e.g., K07515 belonged to five categories, namely, carbohydrate, lipid, amino acid, and other amino acid metabolism and xenobiotic biodegradation and metabolism).

The results of HHMER search showed that 553 proteins families were related to Pfam. The most dominant protein family was phage-related (54 families), followed by type-III-secretion-related proteins (five families). We detected EspB, a type-III secreted pore-forming protein of enteropathogenic *E. coli* (EPEC), which is essential for EPEC pathogenesis (10), indicating that *E. coli* O157:H7 ATCC 43889 functions as an EPEC and EHEC (Table S2).

GO analysis classification was based on annotation from the *E. coli* best-hit gene models (*E* value ≤ 10^-5^), and the number of classified genes of acid-adapted *E. coli* is shown in Fig. S2. The most functional annotated GO was cellular process (2820 CDSs) under Biological Process (BP), followed by metabolic process (2567 CDSs) under BP, cell part under (2514 CDSs) Cellular Component, single-organism process (2403 CDSs) under BP, and binding (2334 CDSs) under Molecular Function.

**Supplementary Tables and Figure legends**

Supplementary Table S1. Genomic features of ATCC 43889.

| Item | Value | | |
| --- | --- | --- | --- |
|  | Total | Chromosome | Plasmid |
| Mean subread length (bp) | 7,813 |  |  |
| N50 (bp) | 11,372 |  |  |
| Total number of bases | 785,423,132 |  |  |
| Number of reads | 100,521 |  |  |
| Number of contig | 2 | 1 | 1 |
| Total genome length (bp) | 5,660,288 | 5,567,434 | 92,854 |
| G+ C contents (%) | 50.5 | 50.5 | 47.8 |
| Number of coding sequence | 5,327 | 5,238 | 89 |
| Number of tRNA | 107 | 107 | 0 |
| Number of rRNA | 22 | 22 | 0 |

Supplementary Table S2. Pfam analysis on whole genome annotation.

| Pfam_ID | Numbers of Pfam | Discription |  | Pfam_ID | Numbers of Pfam | Discription |
| --- | --- | --- | --- | --- | --- | --- |
| PF00089.20 | 1 | Trypsin |  | PF05130.6 | 1 | FlgN protein |
| PF00092.22 | 1 | von Willebrand factor type A domain |  | PF05135.7 | 2 | Phage gp6-like head-tail connector protein |
| PF00128.18 | 1 | Alpha amylase, catalytic domain |  | PF05136.7 | 6 | Phage portal protein, lambda family |
| PF00196.13 | 1 | Bacterial regulatory proteins, luxR family |  | PF05137.7 | 1 | Fimbrial assembly protein (PilN) |
| PF00239.15 | 2 | Resolvase, N terminal domain |  | PF05145.6 | 1 | Putative ammonia monooxygenase |
| PF00310.1 | 1 | Glutamine amidotransferases class-II |  | PF05170.8 | 1 | AsmA family |
| PF00419.14 | 2 | Fimbrial protein |  | PF05193.15 | 1 | Peptidase M16 inactive domain |
| PF00486.22 | 2 | Transcriptional regulatory protein, C terminal |  | PF05269.5 | 2 | Bacteriophage CII protein |
| PF00534.14 | 1 | Glycosyl transferases group 1 |  | PF05354.5 | 2 | Phage Head-Tail Attachment |
| PF00583.18 | 1 | Acetyltransferase (GNAT) family |  | PF05358.5 | 5 | DicB protein |
| PF00589.16 | 5 | Phage integrase family |  | PF05368.7 | 1 | NmrA-like family |
| PF00665.20 | 24 | Integrase core domain |  | PF05406.9 | 2 | WGR domain |
| PF00675.14 | 1 | Insulinase (Peptidase family M16) |  | PF05433.9 | 1 | Glycine zipper 2TM domain |
| PF00723.1 | 1 | Glycosyl hydrolases family 15 |  | PF05489.6 | 1 | Phage Tail Protein X |
| PF00753.21 | 1 | Metallo-beta-lactamase superfamily |  | PF05521.5 | 1 | Phage head-tail joining protein |
| PF00795.1 | 1 | Carbon-nitrogen hydrolase |  | PF05534.6 | 1 | HicB family |
| PF00805.16 | 4 | Pentapeptide repeats (8 copies) |  | PF05594.8 | 1 | Haemagluttinin repeat |
| PF00816.15 | 1 | H-NS histone family |  | PF05666.5 | 1 | Fels-1 Prophage Protein-like |
| PF00847.14 | 1 | AP2 domain |  | PF05717.7 | 5 | IS66 Orf2 like protein |
| PF00872.12 | 5 | Transposase, Mutator family |  | PF05762.8 | 1 | VWA domain containing CoxE-like protein |
| PF00877.13 | 3 | NlpC/P60 family |  | PF05766.6 | 4 | Bacteriophage Lambda NinG protein |
| PF00892.14 | 2 | EamA-like transporter family |  | PF05772.6 | 1 | NinB protein |
| PF00959.13 | 2 | Phage lysozyme |  | PF05802.5 | 1 | Enterobacterial EspB protein |
| PF01012.15 | 1 | Electron transfer flavoprotein domain |  | PF05840.7 | 2 | Bacteriophage replication gene A protein (GPA) |
| PF01062.15 | 1 | Bestrophin, RFP-TM, chloride channel |  | PF05857.5 | 1 | TraX protein |
| PF01075.11 | 1 | Glycosyltransferase family 9 (heptosyltransferase) |  | PF05869.5 | 1 | DNA N-6-adenine-methyltransferase (Dam) |
| PF01118.18 | 1 | Semialdehyde dehydrogenase, NAD binding domain |  | PF05876.6 | 7 | Phage terminase large subunit (GpA) |
| PF01136.13 | 1 | Peptidase family U32 |  | PF05887.5 | 1 | Procyclic acidic repetitive protein (PARP) |
| PF01168.14 | 1 | Alanine racemase, N-terminal domain |  | PF05929.5 | 2 | Phage capsid scaffolding protein (GPO) serine peptidase |
| PF01261.18 | 1 | Xylose isomerase-like TIM barrel |  | PF05930.6 | 4 | Prophage CP4-57 regulatory protein (AlpA) |
| PF01292.14 | 1 | Prokaryotic cytochrome b561 |  | PF05939.7 | 4 | Phage minor tail protein |
| PF01337.12 | 1 | Barstar (barnase inhibitor) |  | PF05944.6 | 1 | Phage small terminase subunit |
| PF01370.15 | 1 | NAD dependent epimerase/dehydratase family |  | PF05954.5 | 2 | Phage late control gene D protein (GPD) |
| PF01381.16 | 4 | Helix-turn-helix |  | PF06064.5 | 2 | Host-nuclease inhibitor protein Gam |
| PF01385.13 | 2 | Probable transposase |  | PF06066.5 | 1 | SepZ |
| PF01402.15 | 2 | Ribbon-helix-helix protein, copG family |  | PF06069.5 | 6 | PerC transcriptional activator |
| PF01442.12 | 1 | Apolipoprotein A1/A4/E domain |  | PF06120.5 | 2 | Tail length tape measure protein |
| PF01464.14 | 2 | Transglycosylase SLT domain |  | PF06141.5 | 3 | Phage minor tail protein U |
| PF01497.12 | 1 | Periplasmic binding protein |  | PF06158.5 | 2 | Helix-destabilising protein |
| PF01527.14 | 24 | Transposase |  | PF06222.5 | 1 | Phage tail assembly chaperone |
| PF01547.19 | 1 | Bacterial extracellular solute-binding protein |  | PF06223.6 | 5 | Minor tail protein T |
| PF01609.15 | 5 | Transposase DDE domain |  | PF06228.7 | 1 | Haem utilisation ChuX/HutX |
| PF01613.12 | 1 | Flavin reductase like domain |  | PF06290.5 | 1 | Plasmid SOS inhibition protein (PsiB) |
| PF01679.11 | 1 | Proteolipid membrane potential modulator |  | PF06291.5 | 2 | Bor protein |
| PF01734.1 | 1 | Patatin-like phospholipase |  | PF06301.5 | 1 | Bacteriophage lambda Kil protein |
| PF01797.10 | 2 | Transposase IS200 like |  | PF06322.5 | 1 | Phage NinH protein |
| PF01835.13 | 1 | MG2 domain |  | PF06323.5 | 4 | Phage antitermination protein Q |
| PF01844.17 | 4 | HNH endonuclease |  | PF06530.6 | 5 | Phage antitermination protein Q |
| PF01923.12 | 1 | Cobalamin adenosyltransferase |  | PF06763.5 | 3 | Prophage minor tail protein Z (GPZ) |
| PF01925.13 | 1 | Sulfite exporter TauE/SafE |  | PF06791.7 | 6 | Prophage tail length tape measure protein |
| PF01928.15 | 1 | CYTH domain |  | PF06805.6 | 8 | Bacteriophage lambda tail assembly protein I |
| PF01943.11 | 2 | Polysaccharide biosynthesis protein |  | PF06812.6 | 1 | ImpA, N-terminal, type VI secretion system |
| PF01963.11 | 1 | TraB family |  | PF06820.6 | 12 | Putative prophage tail fibre C-terminus |
| PF02036.11 | 2 | SCP-2 sterol transfer family |  | PF06891.5 | 1 | P2 phage tail completion protein R (GpR) |
| PF02061.10 | 1 | Lambda Phage CIII |  | PF06894.5 | 5 | Bacteriophage lambda tail assembly chaperone, TAC, protein G |
| PF02090.9 | 1 | Salmonella surface presentation of antigen gene type M protein |  | PF06952.5 | 1 | PsiA protein |
| PF02129.1 | 1 | X-Pro dipeptidyl-peptidase (S15 family) |  | PF06977.5 | 2 | SdiA-regulated |
| PF02195.12 | 1 | ParB-like nuclease domain |  | PF06992.5 | 3 | Replication protein P |
| PF02325.11 | 1 | YGGT family |  | PF06995.5 | 2 | Phage P2 GpU |
| PF02368.12 | 4 | Bacterial Ig-like domain (group 2) |  | PF07152.6 | 1 | YaeQ protein |
| PF02387.9 | 1 | IncFII RepA protein family |  | PF07201.5 | 2 | HrpJ-like domain |
| PF02413.11 | 7 | Caudovirales tail fibre assembly protein, lambda gpK |  | PF07361.5 | 1 | Cytochrome b562 |
| PF02452.11 | 1 | PemK-like, MazF-like toxin of type II toxin-antitoxin system |  | PF07437.5 | 1 | YfaZ precursor |
| PF02517.10 | 1 | CAAX protease self-immunity |  | PF07450.5 | 2 | Formate hydrogenlyase maturation protein HycH |
| PF02563.10 | 1 | Polysaccharide biosynthesis/export protein |  | PF07455.5 | 2 | Phage polarity suppression protein (Psu) |
| PF02627.1 | 1 | Carboxymuconolactone decarboxylase family |  | PF07471.6 | 4 | Phage DNA packaging protein Nu1 |
| PF02635.1 | 1 | DsrE/DsrF-like family |  | PF07669.5 | 1 | Eco57I restriction-modification methylase |
| PF02661.12 | 1 | Fic/DOC family |  | PF07679.10 | 1 | Immunoglobulin I-set domain |
| PF02687.15 | 1 | FtsX-like permease family |  | PF07690.10 | 1 | Major Facilitator Superfamily |
| PF02690.9 | 1 | Na+/Pi-cotransporter |  | PF07703.8 | 1 | Alpha-2-macroglobulin family N-terminal region |
| PF02831.9 | 1 | gpW |  | PF07715.9 | 1 | TonB-dependent Receptor Plug Domain |
| PF02924.8 | 1 | Bacteriophage lambda head decoration protein D |  | PF07719.11 | 1 | Tetratricopeptide repeat |
| PF03006.14 | 1 | Haemolysin-III related |  | PF07728.8 | 1 | AAA domain (dynein-related subfamily) |
| PF03050.8 | 17 | Transposase IS66 family |  | PF07906.7 | 4 | ShET2 enterotoxin, N-terminal region |
| PF03176.9 | 1 | MMPL family |  | PF07947.8 | 1 | YhhN family |
| PF03237.9 | 1 | Terminase-like family |  | PF07977.7 | 1 | FabA-like domain |
| PF03245.7 | 10 | Bacteriophage Rz lysis protein |  | PF07978.7 | 1 | NIPSNAP |
| PF03278.7 | 3 | IpaB/EvcA family |  | PF08238.6 | 1 | Sel1 repeat |
| PF03288.10 | 1 | Poxvirus D5 protein-like |  | PF08242.6 | 1 | Methyltransferase domain |
| PF03313.9 | 1 | Serine dehydratase alpha chain |  | PF08271.6 | 1 | TFIIB zinc-binding |
| PF03354.9 | 1 | Phage Terminase |  | PF08273.6 | 1 | Zinc-binding domain of primase-helicase |
| PF03374.8 | 1 | Phage antirepressor protein KilAC domain |  | PF08291.5 | 1 | Peptidase M15 |
| PF03400.7 | 2 | IS1 transposase |  | PF08298.5 | 1 | PrkA AAA domain |
| PF03432.8 | 1 | Relaxase/Mobilisation nuclease domain |  | PF08346.6 | 1 | AntA/AntB antirepressor |
| PF03589.7 | 3 | Antitermination protein |  | PF08348.5 | 1 | YheO-like PAS domain |
| PF03592.10 | 4 | Terminase small subunit |  | PF08400.4 | 12 | Prophage tail fibre N-terminal |
| PF03621.7 | 1 | MbtH-like protein |  | PF08450.6 | 1 | SMP-30/Gluconolaconase/LRE-like region |
| PF03692.9 | 1 | Putative zinc- or iron-chelating domain |  | PF08486.4 | 1 | Stage II sporulation protein |
| PF03734.8 | 1 | L,D-transpeptidase catalytic domain |  | PF08891.5 | 1 | YfcL protein |
| PF03741.10 | 2 | Integral membrane protein TerC family |  | PF09003.4 | 3 | Bacteriophage lambda integrase, N-terminal domain |
| PF03747.8 | 1 | ADP-ribosylglycohydrolase |  | PF09048.4 | 1 | Cro |
| PF03773.7 | 1 | Predicted permease |  | PF09143.4 | 1 | AvrPphF-ORF-2 |
| PF03783.8 | 1 | Curli production assembly/transport component CsgG |  | PF09392.4 | 3 | Type III secretion needle MxiH, YscF, SsaG, EprI, PscF, EscF |
| PF03797.13 | 1 | Autotransporter beta-domain |  | PF09480.4 | 1 | Type III secretion system protein PrgH-EprH (PrgH) |
| PF03837.8 | 3 | RecT family |  | PF09485.4 | 1 | CRISPR-associated protein Cse2 (CRISPR_cse2) |
| PF03864.9 | 1 | Phage major capsid protein E |  | PF09588.4 | 4 | Conserved hypothetical protein (Lin0512_fam) |
| PF03869.8 | 1 | Arc-like DNA binding domain |  | PF09621.4 | 1 | Type III secretion system regulator (LcrR) |
| PF03881.8 | 1 | Fructosamine kinase |  | PF09669.4 | 1 | Phage regulatory protein Rha (Phage_pRha) |
| PF03916.8 | 1 | Polysulphide reductase, NrfD |  | PF09684.4 | 1 | Phage tail protein (Tail_P2_I) |
| PF03942.9 | 1 | DTW domain |  | PF09691.4 | 1 | Type II secretion system pilotin lipoprotein (PulS_OutS) |
| PF03969.10 | 1 | AFG1-like ATPase |  | PF09695.4 | 1 | Bacterial protein of unknown function (YtfJ_HI0045) |
| PF04011.6 | 1 | LemA family |  | PF09718.4 | 1 | Lambda phage tail tape-measure protein (Tape_meas_lam_C) |
| PF04012.6 | 1 | PspA/IM30 family |  | PF10145.3 | 2 | Phage-related minor tail protein |
| PF04225.6 | 1 | Opacity-associated protein A LysM-like domain |  | PF10543.3 | 3 | ORF6N domain |
| PF04228.7 | 1 | Putative neutral zinc metallopeptidase |  | PF10547.3 | 2 | P22_AR N-terminal domain |
| PF04266.8 | 1 | ASCH domain |  | PF10548.3 | 1 | P22AR C-terminal domain |
| PF04309.6 | 1 | Glycerol-3-phosphate responsive antiterminator |  | PF10549.3 | 1 | ORF11CD3 domain |
| PF04346.6 | 1 | Ethanolamine utilisation protein, EutH |  | PF10554.3 | 1 | Ash protein family |
| PF04380.7 | 1 | Membrane fusogenic activity |  | PF10685.3 | 3 | Stress-induced bacterial acidophilic repeat motif |
| PF04434.11 | 1 | SWIM zinc finger |  | PF10722.3 | 1 | Putative bacterial sensory transduction regulator |
| PF04492.7 | 4 | Bacteriophage replication protein O |  | PF10723.3 | 1 | Replication regulatory protein RepB |
| PF04550.6 | 1 | Phage holin family 2 |  | PF10743.3 | 1 | Regulatory phage protein cox |
| PF04586.11 | 2 | Caudovirus prohead serine protease |  | PF10784.3 | 1 | Plasmid stability protein |
| PF04717.6 | 4 | Type VI secretion system, phage-baseplate injector |  | PF11106.2 | 1 | Exopolysaccharide production protein YjbE |
| PF04748.7 | 1 | Divergent polysaccharide deacetylase |  | PF11112.2 | 1 | Pyocin activator protein PrtN |
| PF04752.6 | 1 | ChaC-like protein |  | PF11439.2 | 1 | Type III secretion system filament chaperone CesA |
| PF04809.7 | 1 | HupH hydrogenase expression protein, C-terminal conserved region | | PF11740.2 | 1 | Plasmid replication region DNA-binding N-term |
| PF04851.9 | 1 | Type III restriction enzyme, res subunit |  | PF12421.2 | 10 | Fibronectin type III protein |
| PF04860.6 | 4 | Phage portal protein |  | PF12486.2 | 1 | Type VI secretion system, EvfB, or VasL |
| PF04865.8 | 1 | Baseplate J-like protein |  | PF12568.2 | 1 | Acetyltransferase (GNAT) domain |
| PF04888.6 | 1 | Secretion system effector C (SseC) like family |  | PF12695.1 | 2 | Alpha/beta hydrolase family |
| PF04932.9 | 1 | O-Antigen ligase |  | PF12697.1 | 2 | Alpha/beta hydrolase family |
| PF04965.8 | 2 | Gene 25-like lysozyme |  | PF12728.1 | 2 | Helix-turn-helix domain |
| PF04971.6 | 10 | Bacteriophage P21 holin S |  | PF12759.1 | 2 | InsA C-terminal domain |
| PF04984.8 | 2 | Phage tail sheath protein |  | PF12790.1 | 1 | Type VI secretion lipoprotein, VasD, EvfM, TssJ, VC_A0113 |
| PF04985.8 | 2 | Phage tail tube protein FII |  | PF12796.1 | 2 | Ankyrin repeats (3 copies) |
| PF05016.8 | 1 | ParE toxin of type II toxin-antitoxin system, parDE |  | PF12844.1 | 1 | Helix-turn-helix domain |
| PF05063.8 | 1 | MT-A70 |  | PF12846.1 | 1 | AAA-like domain |
| PF05099.7 | 2 | Tellurite resistance protein TerB |  | PF12870.1 | 1 | Domain of unknown function (DUF4878) |
| PF05100.6 | 7 | Phage minor tail protein L |  | PF12919.1 | 1 | TcdA/TcdB catalytic glycosyltransferase domain |
| PF05125.6 | 1 | Phage major capsid protein, P2 family |  | PF13007.1 | 1 | Transposase C of IS166 homeodomain |
| Total | 553 |  |  |  |  |  |

Supplementary Table S3. Statistics of RNA-seq alignment of acid-adapted cells and non-adapted cells when mapped to the reference genome.

| Mapping to the reference genome | Acid-adapted | | Non-adapted | |
| --- | --- | --- | --- | --- |
|  | Number | Percentage | Number | Percentage |
| Total read based | 5,441,879,887 | 100 | 6,133,614,875 | 100 |
| Total read pairs | 54,247,194 | 100 | 61,118,940 | 100 |
| Mapped reads^a)^ | 39,955,018 | 73.65 | 44,085,482 | 72.13 |
| Read that failed to align | 12,345,066 | 22.76 | 14,472,760 | 23.68 |
| Suppressed multiple mapped reads^b)^ | 147,110 | 3.59 | 2,560,698 | 1.19 |
| G + C content |  | 51.63 |  | 51.84 |

^a)^ reads with at least one reported alignment^; b)^ reads with alignment suppressed due to –m option

Supplementary Table S4. Differentially expressed genes downregulated in acid-adapted *E. coli* O157:H7 ATCC 43889 compared to corresponding non-adapted using RNA sequencing after rank 16**.**

| Regulation | Rank | length (bp) | Fold change log_2_ ratio | p-value | Gene symbol | Product |
| --- | --- | --- | --- | --- | --- | --- |
| Down | 16 | 216 | -2.57 | 0.06 | *RS26920* | Cell division protein ZapA |
|  | 17 | 180 | -2.57 | 0.03 | *RS22615* | GhoT/OrtT family toxin |
|  | 18 | 1158 | -2.57 | 0.03 | *intS_5* | Putative prophage CPS-53 integrase |
|  | 19 | 956 | -2.51 | 0.04 | *RS16000* | DUF2713 family protein |
|  | 20 | 263 | -2.47 | 0.06 | *RS12915* | Hypothetical protein |
|  | 21 | 165 | -2.46 | 0.04 | *RS03225* | DUF1482 family protein |
|  | 22 | 549 | -2.45 | 0.04 | *RS14560* | DUF2686 family protein |
|  | 23 | 209 | -2.43 | 0.04 | *hha* | Hemolysin expression-modulating protein Hha |
|  | 24 | 233 | -2.35 | 0.06 | *RS22650* | Type III secretion protein |
|  | 25 | 603 | -2.30 | 0.06 | *RS19855* | Hypothetical protein |
|  | 26 | 402 | -2.29 | 0.06 | *RS15185* | DUF2170 family protein |
|  | 27 | 189 | -2.28 | 0.06 | *RS03225* | Cell division inhibitor |
|  | 28 | 1236 | -2.24 | 0.06 | *chuR_1* | Anaerobic sulfatase-maturating enzyme |
|  | 29 | 221 | -2.21 | 0.07 | *RS28945* | Hypothetical protein |
|  | 30 | 198 | -2.21 | 0.07 | *RS15975* | Hypothetical protein |
|  | 31 | 222 | -2.19 | 0.10 | *RS13045* | Hypothetical protein |
|  | 32 | 876 | -2.18 | 0.07 | *RS00070* | Hypothetical protein |
|  | 33 | 354 | -2.18 | 0.07 | *arsR* | Arsenical resistance operon repressor |
|  | 34 | 90 | -2.15 | 0.08 | *RS28100* | DUF551 domain-containing protein |
|  | 35 | 1059 | -2.15 | 0.08 | *RS22610* | Glycosyl hydrolases family 15 |
|  | 36 | 1599 | -2.14 | 0.08 | *adrB_1* | Putative cyclic-di-GMP phosphodiesterase AdrB |
|  | 37 | 1656 | -2.14 | 0.08 | *atsA* | Arylsulfatase |
|  | 38 | 1545 | -2.13 | 0.08 | *malF* | Maltose transport system permease protein MalF |
|  | 39 | 1137 | -2.13 | 0.08 | *RS25285* | Phage DNA ejection protein |
|  | 40 | 434 | -2.13 | 0.08 | *RS28950* | DUF1380 domain-containing protein |
|  | 41 | 153 | -2.13 | 0.09 | *RS09915* | Hypothetical protein |
|  | 42 | 264 | -2.13 | 0.09 | *RS12915* | Hypothetical protein |
|  | 43 | 186 | -2.13 | 0.09 | *RS18510* | Hypothetical protein |
|  | 44 | 684 | -2.12 | 0.08 | *yhdJ* | DNA adenine methyltransferase YhdJ |
|  | 45 | 537 | -2.12 | 0.08 | *yfdR* | 5'-deoxynucleotidase |
|  | 46 | 888 | -2.09 | 0.09 | *RS14765* | transposase |
|  | 47 | 270 | -2.06 | 0.10 | *RS00060* | Phage tail protein |
|  | 48 | 531 | -2.04 | 0.10 | *RS00685* | Cytochrome B |
|  | 49 | 1104 | -2.03 | 0.10 | *livJ* | Leu/Ile/Val-binding protein precursor |

Supplementary Table S5. Primer sequence for qRT-PCR.

| Gene | Primer | Sequence (5' → 3') | Length (bp) | Tm | GC % | Product length (bp) |
| --- | --- | --- | --- | --- | --- | --- |
| *kdpA* | kdpA-F | TCACGTTATGGGTGCTCGTC | 20 | 60.11 | 55.00 | 115 |
|  | kdpA-R | TGCGCTCCTTCAACGGTATT | 20 | 60.04 | 50.00 |  |
| *bhsA* | bhsA-F | GAGCGGAATCTCTCTCACGG | 20 | 59.97 | 60.00 | 109 |
|  | bhsA-R | GCTTAGGTTTGATGCGCCAG | 20 | 59.90 | 55.00 |  |
| *RS6135* | 6135-F | GGCCAACATAGTGGCGGTA | 19 | 59.78 | 57.89 | 84 |
|  | 6135-R | ACCATGGCTATTCTGACCACC | 21 | 59.79 | 52.38 |  |
| *RS8170* | 8170-F | CAAACCATCGAGGCGGTTCC | 20 | 61.64 | 60.00 | 86 |
|  | 8170-R | GTGACTGCTCTTGCCACCTT | 20 | 60.54 | 55.00 |  |
| *malE* | malE-F | GGGTCTGACCTTCCTGGTTG | 20 | 59.96 | 60.00 | 147 |
|  | malE-R | TTGCTGGTGTCGATGTTGGA | 20 | 59.89 | 50.00 |  |
| *lamB* | lamB-F | GCGTTTAGCGCAGATGGAAG | 20 | 59.97 | 55.00 | 83 |
|  | lamB-R | AGTTATCACGCAGGTTGGCA | 20 | 59.96 | 50.00 |  |
| 16S rRNA | 16s-F | CGTGCTACAATGGCGCATAC | 20 | 60.04 | 55.00 | 83 |
|  | 16s-R | CTCCAATCCGGACTACGACG | 20 | 59.97 | 60.00 |  |

The thermal cycling protocol was as follows: initial denaturation for 10 min at 95°C followed by 40 cycles of 15 s at 95°C, 30 s at 60°C, and 30 s at 72°C. The fluorescence signal was measured at the end of each extension step at 72 °C. After the amplification, a melting curve analysis with a temperature gradient of 0.1°C/s from 60 to 95 °C was performed to confirm that only the specific products were amplified. Finally, the samples were cooled to 20 °C for 10 s.


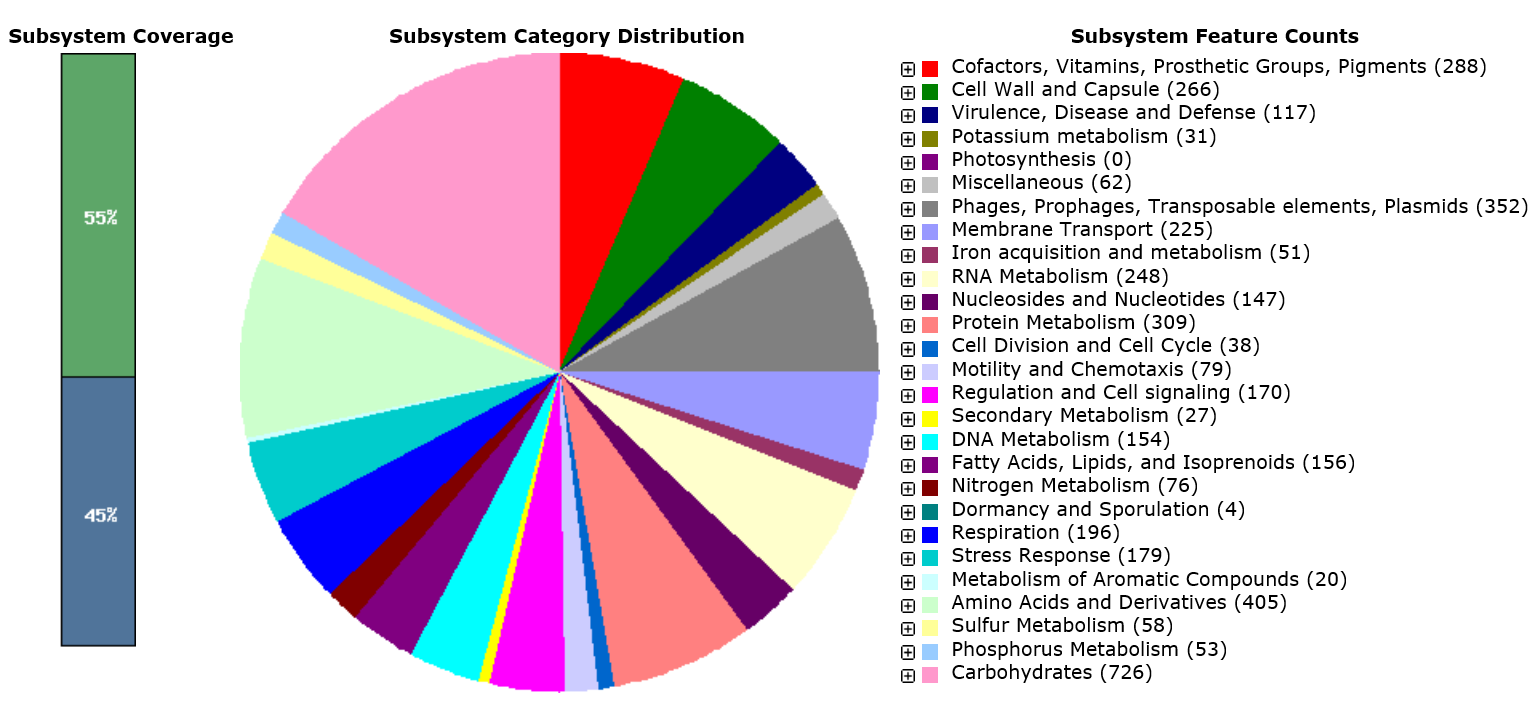


Supplementary Fig S1. Subsystem distribution of SEED and Kyoto Encyclopedia of Genes and Genomes (KEGG). (A) Distribution of genes assigned to 600 SEED subsystems (based on the RAST annotation server). (B) In total, 698 genes were assigned to KEGG distribution (based on BLAST KOALA annotation server).


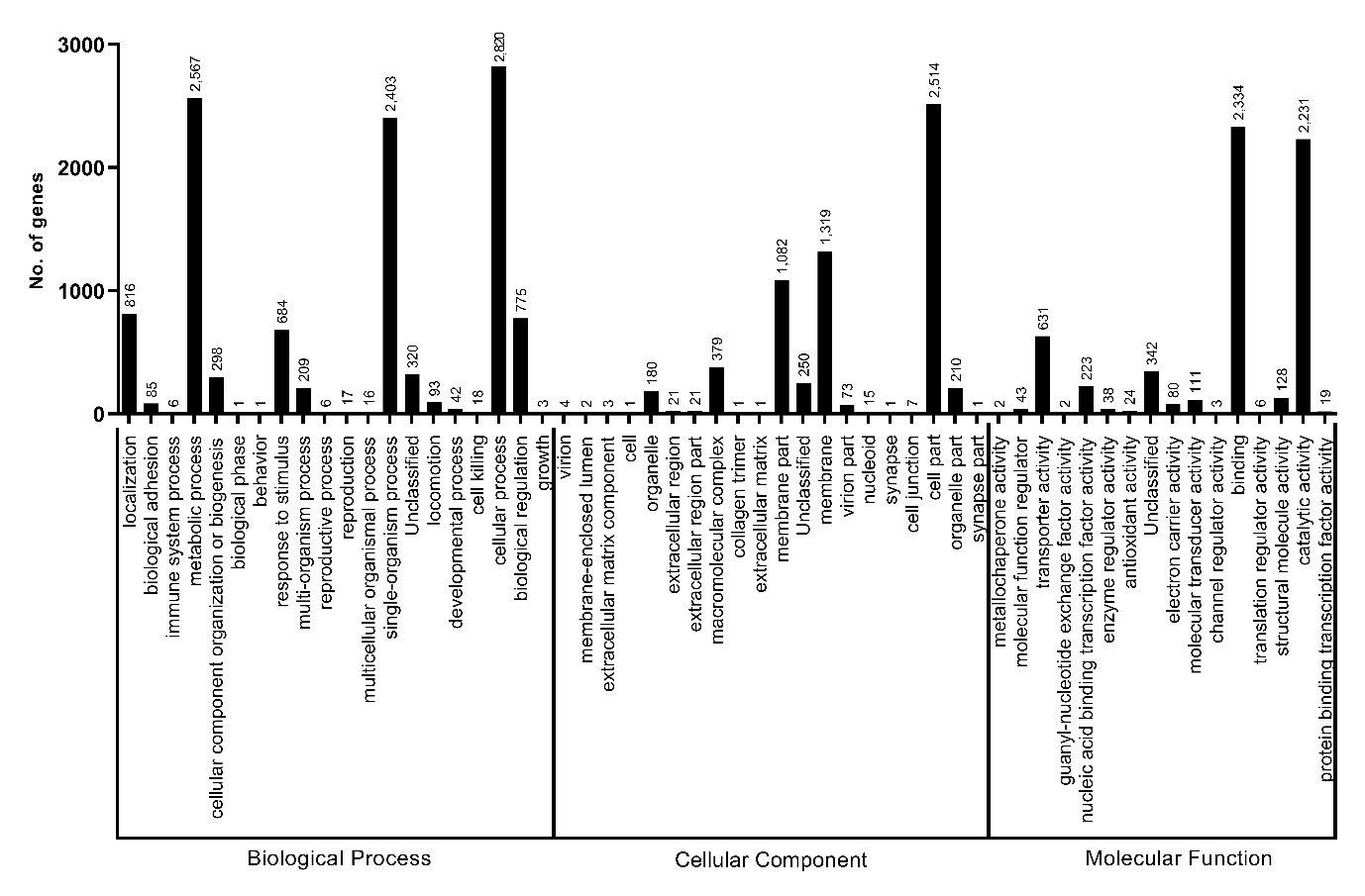


Supplementary Fig S2. Gene Ontology (GO) classification of the 5,327 annotated proteins. GO analysis classifications were based on annotation from the *E. coli* best-hit gene models (*E* value ≤ 10^-5^) and GO analysis search using the BLAST2GO program.

**Supplementary References**

1. Chin C-S, Alexander DH, Marks P, Klammer AA, Drake J, Heiner C, et al. Nonhybrid, finished microbial genome assemblies from long-read SMRT sequencing data. Nat Meth. 2013 Jun;10(6):563–9.

2. Seemann T. Prokka: rapid prokaryotic genome annotation. Bioinforma . 2014 Jul 15;30(14):2068–9.

3. Alikhan N-F, Petty NK, Ben Zakour NL, Beatson SA. BLAST Ring Image Generator (BRIG): simple prokaryote genome comparisons. BMC Genomics. 2011 Aug 8;12:402.

4. Latif H, Li HJ, Charusanti P, Palsson BØ, Aziz RK. A Gapless, Unambiguous Genome Sequence of the Enterohemorrhagic Escherichia coli O157:H7 Strain EDL933. Genome Announc. 2014 Aug 14;2(4):e00821-14.

5. Riley M, Abe T, Arnaud MB, Berlyn MKB, Blattner FR, Chaudhuri RR, et al. Escherichia coli K-12: a cooperatively developed annotation snapshot—2005. Nucleic Acids Res. 2006 Jan 5;34(1):1–9.

6. Eddy SR. Accelerated Profile HMM Searches. PLoS Comput Biol. 2011 Oct 20;7(10):e1002195.

7. Aziz RK, Bartels D, Best AA, DeJongh M, Disz T, Edwards RA, et al. The RAST Server: Rapid Annotations using Subsystems Technology. BMC Genomics. 2008;9(1):75.

8. Kanehisa M, Sato Y, Morishima K. BlastKOALA and GhostKOALA: KEGG Tools for Functional Characterization of Genome and Metagenome Sequences. J Mol Biol. 2016 Feb 22;428(4):726–31.

9. Conesa A, Götz S, García-Gómez JM, Terol J, Talón M, Robles M. Blast2GO: a universal tool for annotation, visualization and analysis in functional genomics research. Bioinformatics. 2005 Sep 15;21(18):3674–6.

10. Tacket CO, Sztein MB, Losonsky G, Abe A, Finlay BB, McNamara BP, et al. Role of EspB in Experimental Human EnteropathogenicEscherichia coli Infection. Infect Immun . 2000 Jun 1;68(6):3689–95.
